# Supplementary figures and images for: Plasmodium SAS4: basal body component of male cell which is dispensable for parasite transmission
Source: Life Sci Alliance. 2022 May 12;5(9):e202101329. doi: 10.26508/lsa.202101329 (PMC9098390; doi:10.26508/lsa.202101329)

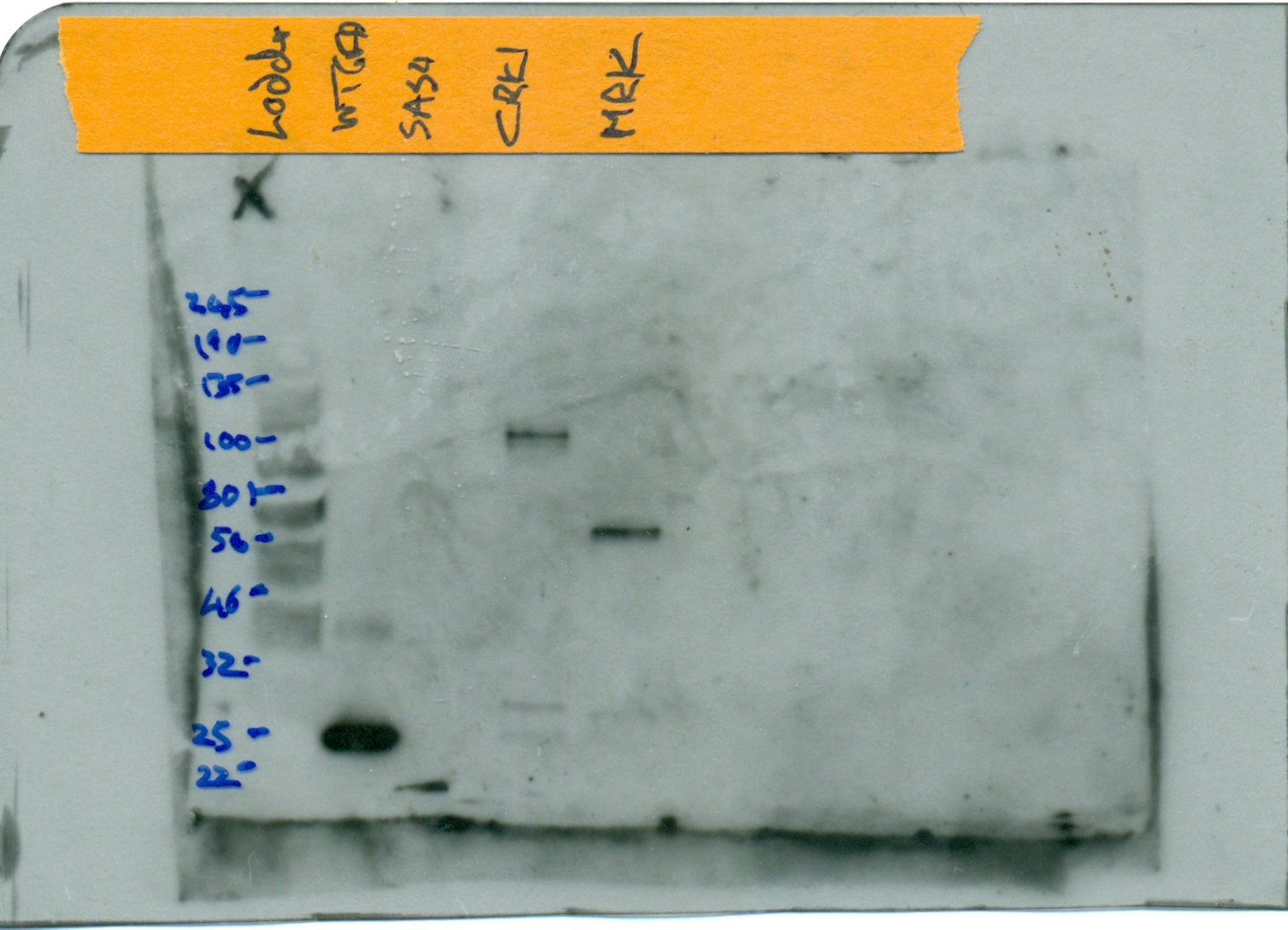

Supplement: Supplementary file 1 [file LSA-2021-01329_SdataFS1.1.jpg]

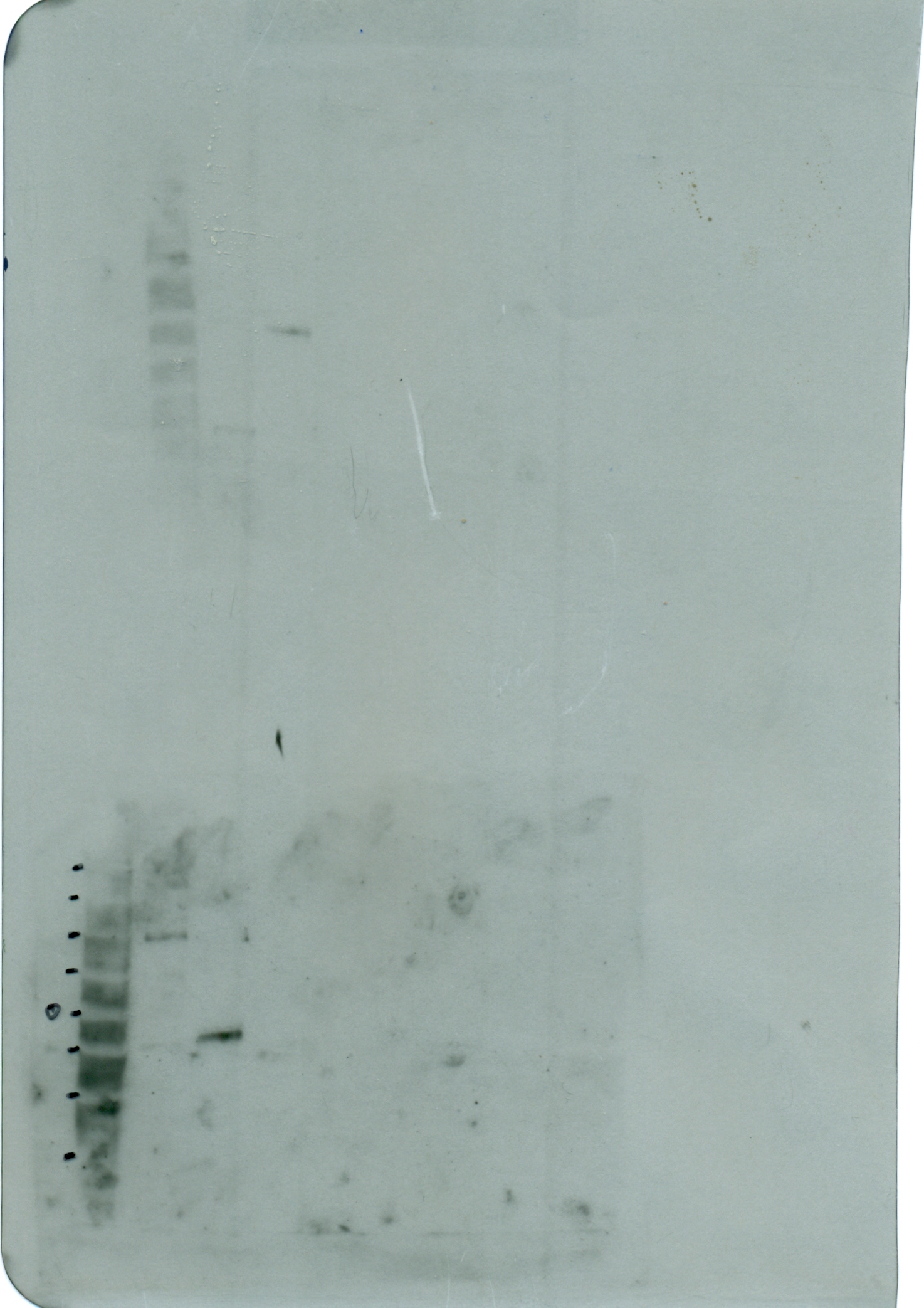

Supplement: Supplementary file 2 [file LSA-2021-01329_SdataFS1.2.jpg]
